# Emergency transcatheter aortic valve replacement for a patient with decompensated severe aortic stenosis accompanied by cardiorenal syndrome: a case report

**DOI:** 10.1186/s12872-018-0791-7

**Published:** 2018-03-20

**Authors:** Hongju Kim, Jung-Hee Lee

**Affiliations:** Division of Cardiology, Yeungnam University Medical Center, Yeungnam University College of Medicine, 3170, Hyeonchung-ro, Nam-gu, Daegu, South Korea

**Keywords:** Transcatheter aortic valve replacement, Heart failure, Cardiorenal syndrome

## Abstract

**Background:**

Severe aortic stenosis (AS) may lead to acute decompensated heart failure resistant to medical treatment. Here, we report a successful emergent transcatheter aortic valve replacement (TAVR) in a patient presenting with decompensated severe AS accompanied by cardiorenal syndrome.

**Case presentation:**

A 82-year-old man presented at our emergency department with aggravated dyspnea. His chest X-ray showed bilateral pulmonary edema, and laboratory examination revealed acute kidney injury. Transthoracic echocardiography (TTE) revealed low-flow, low-gradient AS with decreased left ventricular systolic function. With a diagnosis of acute decompensated heart failure combined with cardiorenal syndrome, we opted to perform emergent TAVR. Ultimately, we successfully performed emergent TAVR using only TTE and 3-D transesophageal echocardiography (TEE) measurements.

**Conclusions:**

This report presents a case of decompensated severe AS accompanied by cardiorenal syndrome that was treated successfully with emergent TAVR. Thus, emergent TAVR using only echocardiography measurements is a feasible and safe option for treating decompensated heart failure accompanied by cardiorenal syndrome the clinical setting.

## Background

Transcatheter aortic valve replacement (TAVR) is an efficient treatment for patients with severe aortic stenosis (AS) at high risk with surgical aortic valve replacement [1]. Severe AS can lead to acute decompensated heart failure and cardiorenal syndrome resistant to medical treatment in some cases. Acute heart failure accompanied by cardiorenal syndrome is the complex physiological, biochemical, and hormonal derangements manifested by worsening renal function during heart failure treatment and diuretic resistance. However, limited data are available to suggest effective treatment options for these patients. Here, we report the successful use of emergent TAVR for a patient presenting with decompensated severe AS accompanied by cardiorenal syndrome.

## Case presentation

**Fig. 1:**
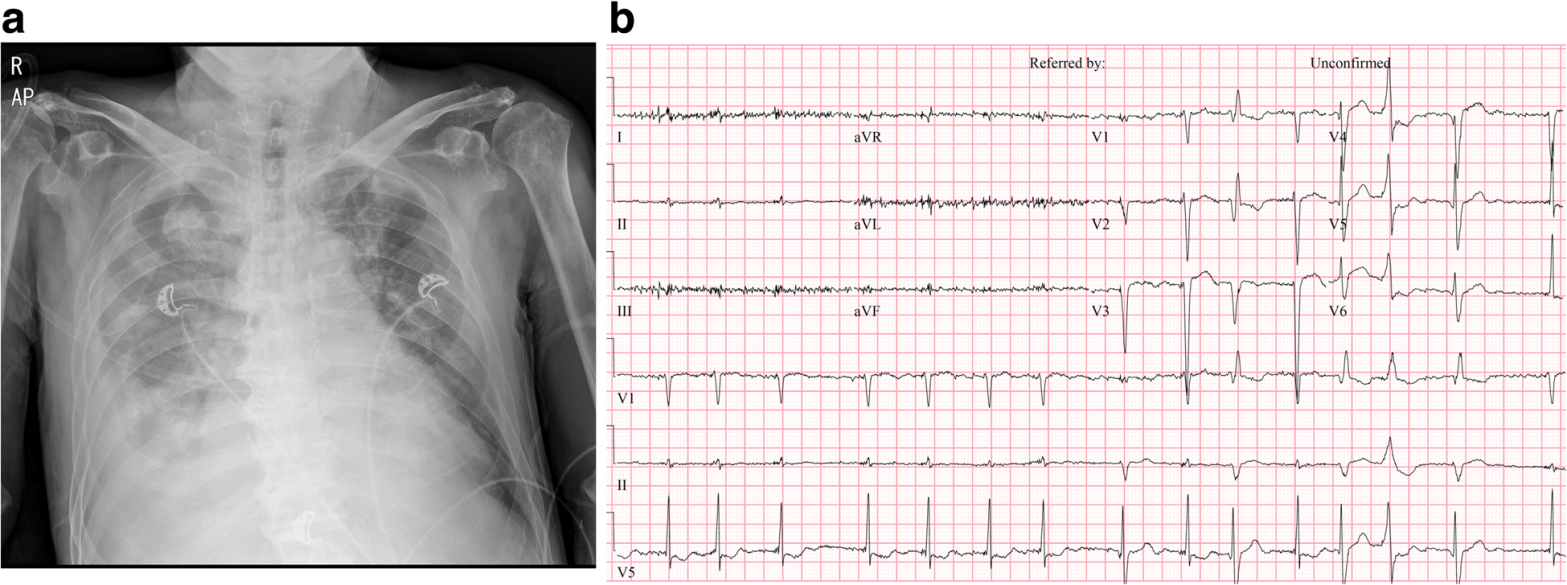
Preoperative findings. **a** Chest X-ray. **b** Electrocardiography

**Fig. 2:**
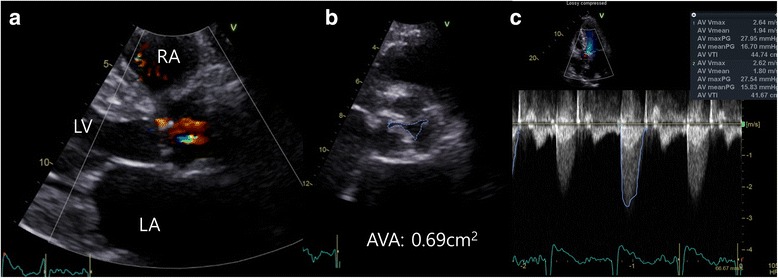
Two-dimensional echocardiography findings. **a** Before the TAVR procedure, echocardiography showed degenerative change in the aortic valve with severe calcification and left ventricular hypertrophy. **b** The aortic valve area was 0.69 cm^2^ based on 2D planimetry. **c** The mean systolic pressure gradient was 16.3 mmHg and aortic valve area was 0.86 cm^2^ based on the continuity eq. RA: right atrium; LV: left ventricle; LA: left atrium

An 82-year-old man presented at our emergency department because of aggravated dyspnea classified as New York Heart Association functional class IV. He has been diagnosed with severe AS 1 year previously, at which time surgical aortic valve replacement was recommended. However, the patient refused open heart surgery at that time. His physical examination showed a blood pressure of 94/56 mmHg, pulse rate of 110 beats per minute, and drowsy mental status. Electrocardiography showed atrial fibrillation with occasional premature ventricular complex, and chest X-ray showed cardiomegaly with bilateral pulmonary edema (Fig. 1). Laboratory examination revealed acute kidney injury, creatinine level of 3.8 mg/dL, and estimated glomerular filtration rate (eGFR) of 16 mL/min/1.73 m^2^. His N-type pro-brain natriuretic peptide level was elevated at 20,774 pg/mL. Transthoracic echocardiography (TTE) revealed low-flow, low-gradient tricuspid aortic valve stenosis (aortic valve area of 0.69 cm^2^ based on 2D planimetry, 0.86 cm^2^ based on the continuity equation, and mean systolic pressure gradient of 16.3 mmHg) with decreased left ventricular (LV) systolic function (LV ejection fraction, 34%) (Fig. 2). Annulus diameter measured by 2D-TTE was 20.6 mm.

With the clinical diagnosis of acute decompensated severe AS accompanied by cardiorenal syndrome, surgical aortic valve replacement was indicated. However, the patient was considered very high-risk candidate for open heart surgery based on the Society of Thoracic Surgeons Predicted Risk of Mortality score (21.153%) [2]. Furthermore, his clinical status had not improved despite intravenous dobutamine infusion with furosemide infusion. Therefore, we ultimately decided to perform emergent TAVR.

**Fig. 3:**
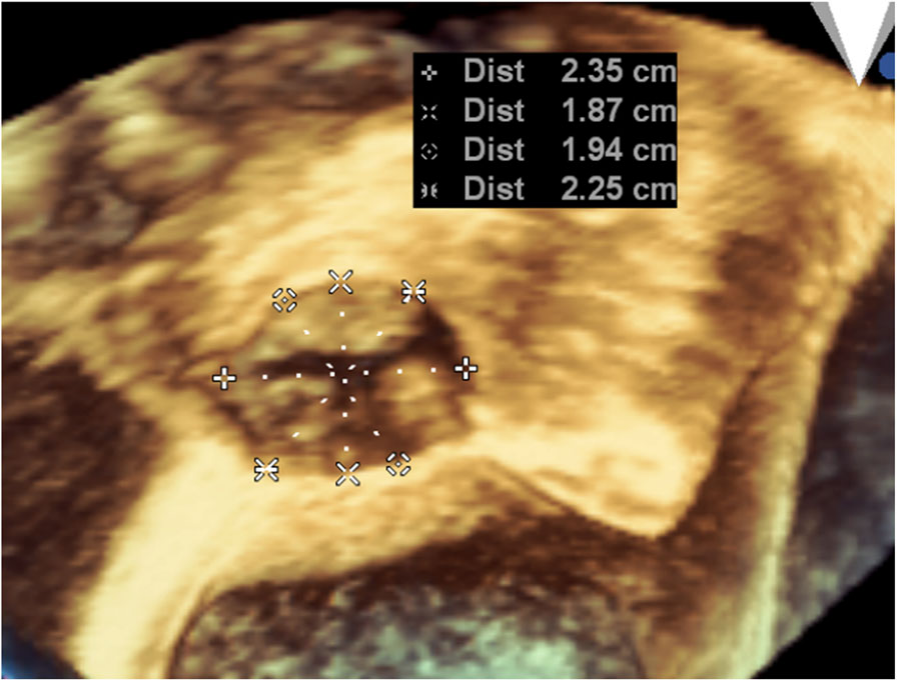
Three-dimensional transesophageal echocardiography findings. Annulus diameter was 23.5 mm

**Fig. 4:**
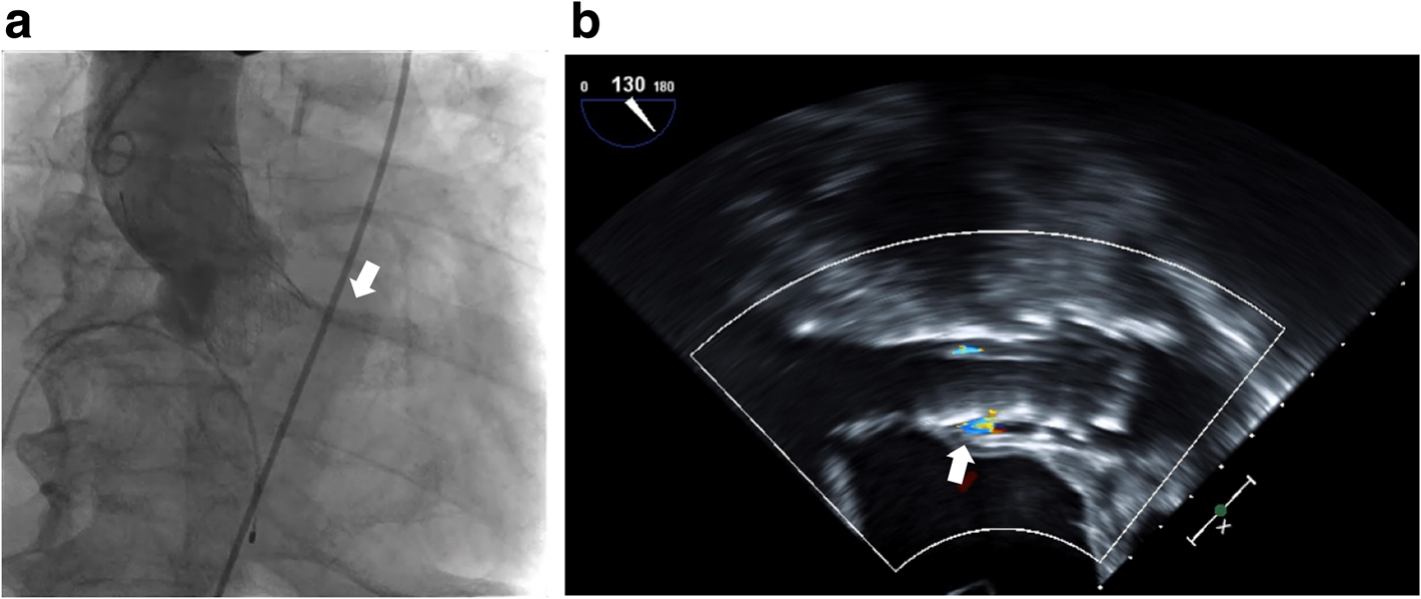
Immediate post-procedural findings. **a** The aortogram revealed mild paravalvular aortic regurgitation (arrow). **b** Transesophageal echocardiography showed mild paravalvular aortic regurgitation (arrow)

The TAVR procedure was performed under general anesthesia. In general, CT should be required to determine the procedure approach and prosthesis size, but it might be dangerous for a patient with decompensated heart failure accompanied by cardiorenal syndrome. Before the TAVR procedure, we attempted to obtain accurate information about annulus diameter by 3D-transesophageal echocardiography (TEE). The TEE annulus diameter measurement of 23.5 mm corresponded to that of the CoreValve transcatheter valve (26-mm; Evolute-R™, Medtronic, NY, USA) (Fig. 3). We punctured the left common femoral artery using the standard percutaneous access techniques, and peripheral angiogram was performed using Judkins Right 4.0 coronary catheter. Fortunately, peripheral angiography showed adequate size of the right iliofemoral artery for vascular access for the CoreValve delivery. Coronary angiography also showed no significant luminal narrowing. Then, we inserted a 26-mm self-expanding CoreValve through the right common femoral artery sheath and deployed it at the aortic valve under fluoroscopic guidance without rapid pacing. The immediate post-procedural aortogram and TEE showed mild paravalvular aortic regurgitation (Fig. 4). The patient’s vital signs were stable during the procedure.

The patient’s dyspnea was dramatically improved after the procedure, and chest X-ray showed marked improvement. The post-procedural TTE revealed improved LV systolic function (LV ejection fraction, 45%) with well-functioning CoreValve (aortic valve area of 2.15 cm^2^ based on the continuity equation and mean systolic pressure gradient of 3.22 mmHg). Urine output was also increased, creatinine level was decreased to 1.4 mg/dL, and eGFR increased to 52 mL/min/1.73 m^2^. He was discharged 7 days after the procedure, and his 3-month follow-up echocardiography showed no significant interval change from his immediate post-procedural findings.

## Discussion and conclusions

There is a high prevalence of severe AS in elderly patients, usually combined with multiple risk factors. In elderly patients with severe AS, surgery is contraindicated in about 30% of cases because of older age and LV dysfunction, which are associated with increased operative risk and a poor late outcome after surgery [3]. TAVR is currently the “gold-standard” treatment for these high-risk elderly patients with severe AS, and the indications have been extended to intermediate-risk patients [4, 5]. Patients with severe AS may experience acute decompensated heart failure accompanied by cardiorenal syndrome that is resistant to medical treatment. Heart failure and renal failure have synergistic effects that increase the adverse outcomes compared to those of either disease alone [6]. The present case report illustrates the effectiveness of emergency TAVR in the setting of decompensated severe AS accompanied by cardiorenal syndrome. Bongiovanni et al. investigated the early outcome of emergency TAVR versus emergency balloon aortic valvuloplasty for the treatment of patients with acute AS and severe decompensation [7]. Emergency TAVR showed high immediate procedural and 30-day mortalities compared with emergency balloon aortic valvuloplasty, but TAVR was associated with a trend toward lower mortality at the 2-year follow-up [7]. Frerker et al. reported outcomes of TAVR as a rescue therapy in patients with cardiogenic shock due to acute decompensated heart failure caused by severe AS and found that 1-year survival did not differ between emergency and elective TAVR among 30-day survivors (89.6% vs. 88.9%) [8]. This finding suggests that emergency TAVR might be a reasonable therapeutic option for patients with acute decompensated severe AS.

Procedural complications following TAVR, such as paravalvular regurgitation, stroke, permanent pacemaker implantation, vascular access injury, and renal failure, are commonly reported as previous literature. Especially, significant postprocedural aortic regurgitation after TAVR observed 10% to 20% is reported one of the strongest independent predictor of mortality [9]. Peri-prosthetic aortic regurgitation result from under-expansion of the prosthesis stent frame, which might be caused by calcification of the annulus or the cusps of the native valve, malposition with too shallow or too deep implantation depth of the prosthesis, or annulus-prosthesis size mismatch [10].

For successful TAVR, multimodality imaging examination, including computed tomography (CT), is necessary to determine the procedure approach and prosthesis size [11, 12]. However, contrast-enhanced CT may aggravate renal impairment in patients with cardiorenal syndrome. A previous study of imaging techniques for the measurement of aortic annulus diameter before TAVR reported that 3D-TEE provided similar results to those obtained by CT [13]. In this case, annulus diameter measured by 2D-TTE was 20.6 mm, which is smaller than that measured by 3D-TEE (23.5 mm). We selected the prosthesis size based on 3D-TEE findings, and we ultimately successfully implanted a CoreValve of adequate size with only mild paravalvular aortic regurgitation. In the clinical setting of severely decompensated patients with severe AS, emergency TAVR can be performed using 3D-TEE imaging, if CT is not possible. This case report is important as it reports on effectiveness of emergent TAVR in the clinical setting of acute decompensated heart failure accompanied by cardiorenal syndrome, which requires immediate decision making. In conclusion, this case report presents an effective strategy for successful emergent TAVR in a patient with decompensated severe AS accompanied by cardiorenal syndrome. Thus, emergent TAVR using only echocardiography measurements is a feasible and safe option for treating decompensated heart failure accompanied by cardiorenal syndrome in the clinical setting.

## Abbreviations

AS: Aortic stenosis
CT: Computed tomography
eGFR: Estimated glomerular filtration rate
LV: Left ventricular
TAVR: Transcatheter aortic valve replacement
TEE: Transesophageal echocardiography
TTE: Transthoracic echocardiography
